# Combination of Platelet expression of PKCepsilon and cardiac troponin-I for early diagnosis of chest pain patients in the emergency department

**DOI:** 10.1038/s41598-019-38624-5

**Published:** 2019-02-14

**Authors:** Cecilia Carubbi, Elena Masselli, Giulia Pozzi, Maria Mattioli, Silvia Martini, Matteo Goldoni, Rosalia Aloe, Gianfranco Cervellin, Marco Vitale, Giuliana Gobbi

**Affiliations:** 1 Department of Medicine and Surgery, University of Parma, Parma, Italy; 2 Division of Cardiology, Azienda Ospedaliero-Universitaria di Parma, Parma, Italy; 3 Protein Phosphorylation Laboratory, Francis Crick Institute, London, United Kingdom; 4 Dipartimento di Biochimica ad Elevata Automazione, Dipartimento Diagnostico, Azienda Ospedaliero-Universitaria di Parma, Parma, Italy; 5 Emergency Department, Azienda Ospedaliero-Universitaria di Parma, Parma, Italy; 6 Sport and exercise medicine center (SEM), University of Parma, Parma, Italy; 7 CoreLab, Azienda Ospedaliero-Universitaria di Parma, University of Parma, Parma, Italy

## Abstract

A rapid differential diagnosis of the clinical conditions underlying chest pain is a relevant clinical issue. Specifically, a fast rule-in or -out of acute myocardial infarction (AMI) is mandatory to improve diagnostic outcome and cost-effectiveness of patient management. We demonstrated that Protein Kinase C (PKC) epsilon is selectively expressed by platelets from AMI patients, accounting for increased platelet activation. Thus, we hypothesized that PKCepsilon-expressing platelets may represent a pathophysiological marker of AMI that could be utilized in combination with troponin-I, the conventional marker of cardiac injury, to add diagnostic information in chest pain workup. In 94 chest pain patients consecutively admitted to Parma University Hospital, we tested the diagnostic performance of flow-cytometric detection of PKCepsilon expressing platelets in discriminating AMI vs. non-AMI conditions. We demonstrated that PKCepsilon-expressing platelets were significantly higher in patients with AMI. Flow cytometry detection of PKCepsilon-expressing platelets showed high sensitivity and specificity (87.5% and 84.4%, respectively) and good diagnostic accuracy (AUC: 0.875). The combination of PKCepsilon expressing platelets and cardiac troponin clearly discriminates patients with 100% and 0% of probability to be affected by AMI. Overall, we highlighted a dual marker strategy potentially useful for a rapid rule-in or -out of myocardial infarction in chest pain patients.

## Introduction

Chest pain (CP) is one of the leading complaints in patients seeking medical evaluation at emergency departments (EDs), accounting for up to 10% of the whole ED census^1,2^. Indeed, it is a common symptom of both cardiac and non-cardiac disorders, and the underlying cause may vary from diseases with favorable prognosis to potentially life-threatening conditions, including myocardial infarction, pulmonary embolism, acute aortic syndromes, pneumothorax, or myocarditis. Among them, the majority of ED patients might be suitable for direct discharge from ED, while 15–20% of them have a final diagnosis of acute myocardial infarction (AMI). Precautionary surveillance for CP patients is thus necessary and part of the routine clinical management of this population, leading to long-lasting waiting time, high costs and over-use of ED^3^. Fast MI rule-out often allows direct discharge from ED or helps corroborating alternative diagnosis. Therefore, a timely differential diagnosis of CP patients is a clinical priority^4–8^. Indeed, delays in diagnosis may increase the risk of complications and fatal consequences while, on the other side, unnecessary hospital admissions get worse cost-effectiveness patient management.

Clinical assessment, cardiac troponin assays and electrocardiogram, are the mainstays in triage strategies for the differential diagnosis of AMI, requiring long-lasting clinical observation and further testing. More recently, the use of high sensitivity troponin assay has improved speed and accuracy, developing algorithms that require one or two troponin testing within 1 to 3 hours, thus facilitating clinical decision of patients’ disposition in a very short time^9,10^. Nevertheless, the ability of ruling-out AMI with a single blood draw depends on time of symptoms onset^11^. Moreover, the enhanced clinical sensitivity is accompanied by a reduction in specificity. Therefore, laboratory tests – troponins included– should always be used in conjunction with all other information available to the clinicians^9,10,12–14^. Given this scenario, new tools are eagerly needed to improve the diagnostic algorithm for CP patients and the cost- and time-effectiveness of ED management.

Platelets play a central role in the genesis and propagation of atherothrombosis, the key process underlying type 1 myocardial infarction^11^. Furthermore, platelet hyper-reactive phenotype is a key marker of cardiovascular risk and it represents one of the most common therapeutic target for the prevention of thrombosis^15,16^.

Flow cytometry (FCM) allows a single-shot, multi-parametric characterization of the platelet population. Indeed, FCM detection of platelet reactivity is currently used in clinical practice for the monitoring of anti-aggregation therapies and to assess the thrombotic risk in cardiovascular diseases^17^. Of note, FCM assessment of platelet activation markers has been suggested as a feasible tool for AMI diagnosis^4,18^.

A variety of platelet functions have been associated with protein kinase C (PKC) activity^19^, with different isoforms having a dual control in thrombus formation, balancing the proaggregatory and procoagulant properties of thrombi^20^. We demonstrated that in human platelets, the over-expression of the novel isoform PKCepsilon (PKCe) is associated to a pro-thrombotic phenotype, including AMI and a history of cardiovascular events^21,22^. More in details, we found that this kinase is selectively expressed in platelets of AMI patients, but not in stable coronary artery disease patients and healthy donors. Of note, PKCe-expressing platelets showed an increased expression of platelet activation markers, were hyper-responsive to pro-activatory stimuli and were more prone to adhesion, demonstrating the correlation between PKCe expression and increased platelet activation. In AMI patients, PKCe expression in platelets is increased during the acute event, while it returns negative after 15 days of follow-up. We also demonstrated that the ectopic expression of PKCe involves both mature and young platelets, excluding that the presence of PKCe-positive platelets could be selectively ascribed to newly formed platelets during AMI, rather suggesting that PKCe could be up-regulated before the acute event^21^.

Overall, our previous findings configure platelet expression of PKCe as a marker of the peri-infarctual period, amenable for consideration as a novel diagnostic tool.

In this study, we used flow cytometry to assess PKCe expressing platelets in patients presenting to the emergency department with a complaint of chest pain, testing its diagnostic performance in discriminating patients with or without AMI.

## Results

### Patients’ characteristics

Of the 94 patients consecutively enrolled in this study, 5 patients were excluded because leaving the hospital voluntarily without completing the diagnostic work-up. The baseline characteristics of the remaining 89 patients are shown in Table 1. AMI was diagnosed in 12 (13.5%) out of 89 patients (Supplementary Fig. 1a). Of them, 6 suffered from ST-segment elevation myocardial infarction (STEMI) and 6 from non-ST-segment elevation myocardial infarction (NSTEMI) (Supplementary Fig. 1b). The other adjudicated final diagnoses were unstable angina in 5 patients (6.5%), cardiac causes other than coronary artery disease in 21 patients (27.3%), non-cardiac causes in 28 patients (36.4%), and symptoms of unknown origin in 23 patients (29.8%) (Supplementary Fig. 1c).

**Table 1:** Baseline characteristics of entire patient’s population.

	CP-noAMI	CP-AMI	Statistical analysis
No of cases, *n (%)*	77 (86.5)	12 (13.5)	
**GENDER**			
Male, *n (%)*	49 (63, 6)	8 (66, 6)	n.s (Chi Square)
Female, *n (%)*	28 (36, 4)	4 (33, 4)	
AGE, *median (range)*	72 (24–96)	79 (53–88)	n.s Mann-Whitney/Wilcoxon
RDW, *mean* ± *sd*	14 ± 1	14 ± 1	n.s (Student t test)
MPV, *mean* ± *sd*	9 ± 1	9 ± 1	n.s (Student t test)
GLYCEMIA, *mean* ± *sd*	117 ± 34	146 ± 58	n.s (Mann-Whitney/Wilcoxon)
Time of symptom onset, *mean* ± *sd*	32 ± 59	18 ± 28	n.s (Student t test)
**Pharmacological treatment**			
Pts on anti-PLT drugs, *n (%)*	27 (42.18)	7 (63.6)	n.s (Chi Square)
Pts not on anti-PLT drugs, *n (%)*	37 (57.8)	4 (36.36)	

*n: number; Pts: patients; RDW: Red Cell Distribution Width; MPV: mean platelet volume; sd: standard deviation; n*.*s: no statistical significance*.

In the subset of 53 patients used for triple staining analysis, AMI was 15.1% (with 75% STEMI and 25% NSTEMI), unstable angina 4.4%, cardiac causes other than coronary artery disease 31.1%, non-cardiac causes in 33.3% and symptoms of unknown origin in 31.1% (Supplementary Fig. 1d,f).

Patients with and without final diagnosis of AMI were homogeneous for age, gender, time-of-symptoms-onset distribution. The percentage of patients on anti-platelet drug(s) (ASA, clopidrogel, ticagrerol) was also similar in the two groups. (Tables 1 and 2).

**Table 2:** Baseline characteristics of subset patient’s population.

	CP-noAMI	CP-AMI	Statistical analysis
No of cases, *n (%)*	45 (84.9)	8 (15.1)	
**GENDER**			
Male, *n (%)*	24 (53.3)	5 (62.5)	n.s (Chi Square)
Female, *n (%)*	21 (46.7)	3 (37.5)	
AGE, *median (range)*	72 (29–83)	74 (53–86)	n.s (Mann-Whitney/Wilcoxon)
RDW, *mean* ± *sd*	14 ± 1	13 ± 1	n.s (Student t test)
MPV, *mean* ± *sd*	9 ± 2	8 ± 2	n.s (Student t test)
GLYCEMIA, *mean* ± *sd*	118 ± 37	144 ± 62	n.s (Mann-Whitney/Wilcoxon)
Time of symptom onset, *mean* ± *sd*	25 ± 41	23 ± 31	n.s (Student t test)
**Pharmacological treatment**			
Pts on anti-PLT drugs, *n (%)*	12 (31.6)	3 (42.8)	n.s (Chi Square)
Pts not on anti-PLT drugs, *n (%)*	26 (68.4)	4 (57.2)	

n: number; Pts: patients; RDW: Red Cell Distribution Width; MPV: mean platelet volume; sd: standard deviation; n.s: no statistical significance.

### PKCepsilon expressing platelets are increased in CP patients with AMI

It has been previously reported that PKCepsilon is nearly absent in human normal platelets^23,24^, while its expression has been detected in platelets from patients with AMI^21^. However, these data have been obtained by western blot and RT-PCR based methods, time-consuming techniques that are not appropriate for timely diagnostic purposes in an emergency setting. Therefore, we used a fast FCM assay to detect PKCepsilon expression in platelets of CP patients.

**Figure 1:**
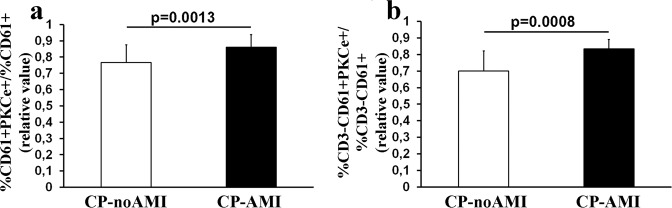
FCM analyses of PKCe-expressing PLTs. Panel (a) PKCe-expressing PLTs in chest patients with (CP-AMI) or without (CP-noAMI) final diagnosis of acute myocardial infarction in entire patient’s population. PKCe-expressing PLTs are reported as CD61^+^PKCe^+^ cell population normalized according to CD61^+^ cells. Data are reported as mean ± SD. Horizontal line represents statistical analysis, performed by Student t-test. Panel (b) PKCe-expressing PLTs in chest patients with (CP-AMI) or without (CP-noAMI) final diagnosis of acute myocardial infarction in the subset of enrolled patients. PKCe-expressing PLTs are reported as CD3^−^CD61^+^PKCe^+^cell population normalized according to CD3^−^CD61^+^ cells. Data are reported as mean ± SD. Horizontal line represents statistical analysis, performed by Student t-test.

The double staining with anti-PKCepsilon and anti-CD61 monoclonal antibodies showed that CP patients with a final diagnosis of myocardial infarction (CP-AMI) display a significantly higher percentage of PKCe-expressing PLTs as compared to CP patients not attributable to AMI (CP-noAMI) (Fig. 1a).

Since during activation platelets tend to aggregate with other blood cells such as lymphocytes, which in turn express PKCepsilon, in a subset of enrolled patients (Table 2), lymphocytes population has been excluded using an immunological gate (Supplementary Fig. 2). Accordingly, as reported in Fig. 1 panel b, it has been confirmed that platelet population expressing PKCepsilon is significantly higher in CP-AMI patients as compared to CP-noAMI patients.

Moreover, no differences have been detected between STEMI and NSTEMI and among subtypes of CP-noAMI patients in double staining, as well as in triple staining (Supplementary Fig. 3). Additionally, no differences were found in terms of PKCe expressing platelets among patients on or off antiplatelet drugs in both CP-AMI and CP-noAMI categories (Supplementary Fig. 4).

### Diagnostic value of PKCepsilon expressing platelets

We investigated the diagnostic accuracy of FCM detection of PKCe-expressing PLTs assessing sensitivity and specificity of double and triple staining analysis.

**Figure 2:**
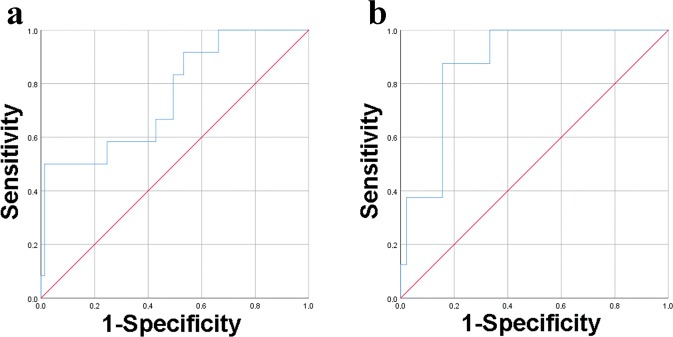
ROC curves of FCM analyses of PKCe-expressing PLTs. Panel (a) ROC curve for PKCe-expressing PLTs analyzed as CD61^+^PKCe^+^/CD61^+^ in the entire patients population. Panel (b) ROC curve for PKCe-expressing PLTs analyzed as CD3^−^CD61^+^PKCe^+^/CD3^−^CD61^+^ in the subset of enrolled patients.

The analysis of PKCe-expressing PLTs as CD61^+^PKCe^+^/CD61^+^ population generates a ROC curve with an AUC of 0.756 [CI of 95%: 0.606–0.907] (Fig. 2a and Table 3). The optimal cut-off value was found at 0.913 at a sensitivity of 50% and a specificity of 98.7%, with a positive predictive value (PPV) of 85.7% and a negative predictive value (NPV) of 92.6% (Table 3). Excluding lymphocytes population, the analysis of PKCe-expressing PLTs (CD3^−^CD61^+^PKCe^+^/CD3^−^CD61^+^) shows an AUC of 0.875 [CI of 95%: 0.774–0.976] (Fig. 2b and Table 3) with an optimal cut-off value at 0.802, reaching a sensitivity of 87.5% and a specificity of 84.4%, with a PPV of 50% and a NPV of 97.7% (Table 3). These data indicate that the analysis of CD61^+^PKCe^+^/CD61^+^ platelet population demonstrates fair diagnostic accuracy, while the analysis of CD3^−^CD61^+^PKCe^+^/CD3^−^CD61^+^ platelet population shows good diagnostic accuracy.

**Table 3:** Area under the curves (AUC), confidential intervals (CI), cut-off values, sensitivity, specificity, positive predictive value (PPV) and negative predictive value (NPV) of FCM detection of PKCe-expressing PLTs.

	AUC [CI 95%]	Cut-off	Sensitivity	Specificity	PPV	NPV
CD61^+^PKCe^+^/ CD61^+^	0.756 [0.606–0.907]	0.913	50%	98.7%	85.7%	92.6%
CD3^−^CD61^+^PKCe^+^/ CD3^−^CD61^+^	0.875 [0.774–0.976]	0.802	87.5%	84.4%	50%	97.7%

Overall, these results suggest that FCM detection of PKCe-expressing PLTs, identified as CD3^−^CD61^+^PKCe^+^/CD3^−^CD61^+^ population, has good diagnostic performance for discriminating patients with myocardial infarction.

Therefore, the subsequent analyses were performed on CD3^−^CD61^+^PKCe^+^/CD3^−^CD61^+^ obtained from this subset patients population.

### Diagnostic value of PKCepsilon expressing platelets in combination with cardiac troponin

Troponin I at presentation was under diagnostic cut-off of 0.06 ug/L in 46 out of 53 (86.8%) patients, including 3 patients with a final diagnosis of MI and 43 patients without final diagnosis of MI. Of note, despite 93.4% (43/46) of these patients did not receive a final diagnosis of myocardial infarction, a second troponin assay has been performed in 56.5% (26/46) of them. These data confirm that serial sampling is required for a safe rule-out and prompt us to investigate whether FCM detection of PKCe-expressing PLTs could add diagnostic benefit to the conventional cardiac troponin assay.

**Figure 3:**
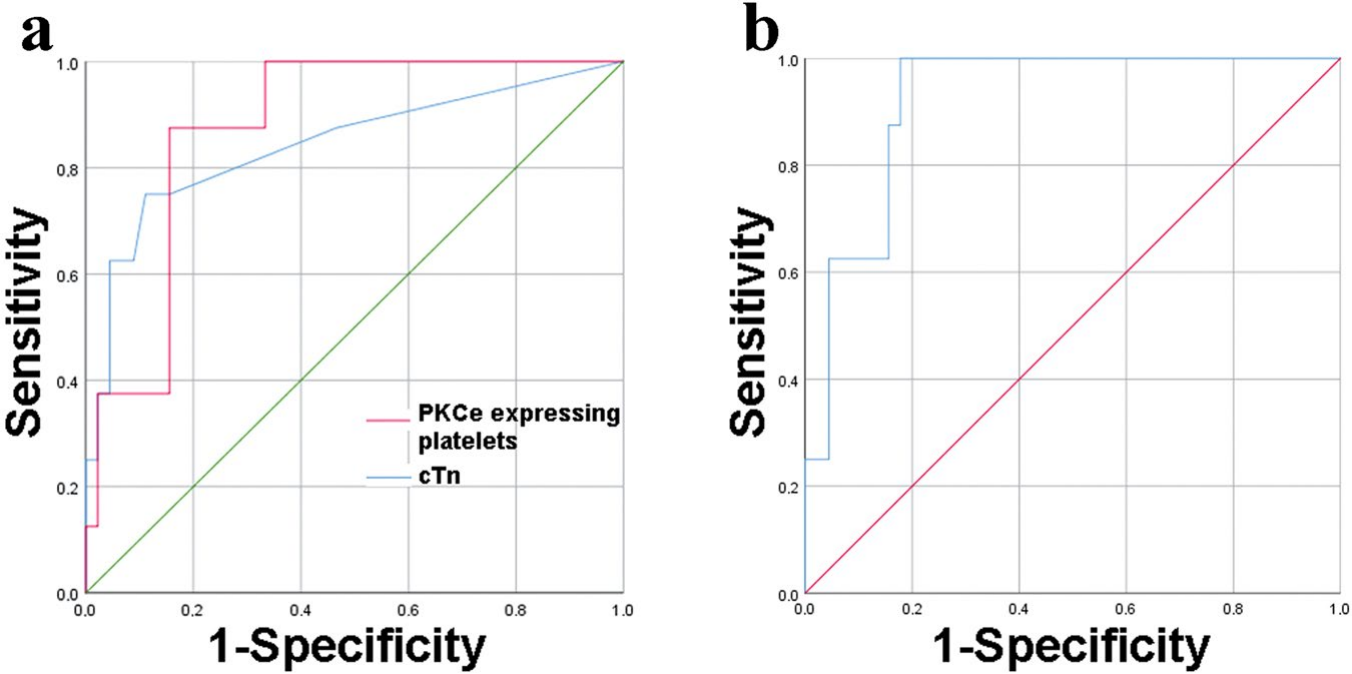
ROC curves of PKCe-expressing PLTs and cTnI. Panel (a) ROC curves, for PKCe-expressing PLTs, analyzed as CD3^−^CD61^+^PKCe^+^/CD3^−^CD61^+^ (red line), and cTnI (blue line) in the subset of enrolled patients. Panel (b) ROC curve of the logistic regression of PKCe-expressing PLTs, analyzed as CD3^−^CD61^+^PKCe^+^/CD3^−^CD61^+^ and cTnI in the subset of enrolled patients.

Firstly, the diagnostic performance of cTnI and PKCe-expressing PLTs has been compared in our subset of patients. Interestingly, the ROC curve of PKCe-expressing PLTs showed that the AUC value is not statistically different from that of cTnI (AUC [95%CI]: 0.875 [0.777–0.976] *vs* 0.843 [0.666–1], respectively) (Fig. 3a and Table 4). Moreover, at the diagnostic cut-off value of 0.06ug/L, cTnI shows a sensitivity of 62.5%, a specificity of 86.6%, with a PPV of 45.4% and a NPV of 92.8% (Table 4). These data may suggest that the analysis of PKCe-expressing PLTs has a better sensitivity with a slight reduction of specificity. (Data on entire patients population analyzing, CD61^+^PKCe^+^/CD61^+^, are reported in Supplementary Fig. 5).

**Table 4:** Area under the curves (AUC), confidential intervals (CI), cut-off values, sensitivity, specificity, positive predictive value (PPV) and negative predictive value (NPV) of PKCe-expressing PLTs, cardiac troponin I (cTnI) and logistic regression model combining PKCe-expressing PLTs and cardiac troponin of enrolled patients.

	AUC [CI 95%]	Cut-off	Sensitivity	Specificity	PPV	NPV
PKCe-expressing PLTs (CD3^−^CD61^+^PKCe^+^/ CD3^−^CD61^+^)	0.875 [0.774–0.976]	0.802	87.5%	84.4%	50%	97.7%
cTnI (ug/L)	0.843 [0.666–1]	0.06	62.5%	86.6%	45.4%	92.8%
Logistic regression of PKCe-expressing PLTs and cTnI	0.922 [0.848–0.997]	0.139	100%	84.4%	53.3%	100%

Secondly, using a Spearmann Correlation test it has been demonstrated that PKCe-expressing PLTs and cardiac troponin levels are two independent factors (p > 0.05). Then, a logistic regression model with these two factors has been applied and, despite a very good diagnostic performance has been achieved, the model fails to significantly improve the diagnostic accuracy of cTnI or PKCe-expressing PLTs alone (AUC [95%CI]: 0.922 [0.848–0.997] *vs* AUC [95%CI]: 0.843 [0.666–1] *or vs* 0.875 [0.777–0.976], respectively) (Fig. 3b and Table 4).

**Figure 4:**
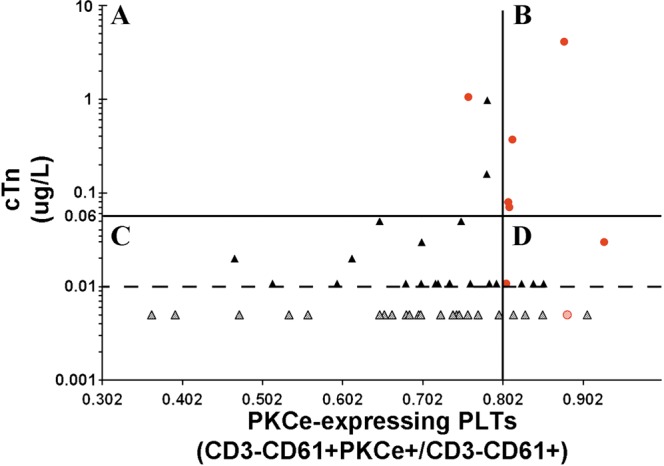
Combination of cut-off points of PKCe-expressing PLTs and cTnI. Chest pain patients of the subset population are represented according to their values of cTnI (Y-axis) and PKCe-expressing PLTs (X-axis) at presentation to ED. The cut-off values of each parameter are represented as black lines and limit of detection of cTnI is represented as dotted black line. Red spots represent CP-AMI, black triangles represent CP-noAMI patients, and patients with undetectable values of cTnI are represented as dotted spots and triangles.

Finally, cTnI and PKCe-expressing PLTs have been combined using simultaneously the two cut-off points as discriminating values, and our data show that patients with both parameters exceeding the cut-off values had a 100% of probability to be affected by AMI; conversely, patients with both parameters under cut-off values had 0% of probability to be hit by AMI (Fig. 4).

## Discussion

In this study, the diagnostic value of PKCe-expressing PLTs in patients admitted to ED because of chest pain has been explored. It has been found that the population of platelets expressing PKCe is significantly higher in patients with a final diagnosis of AMI than in patients with chest pain due to other causes. This result, along with our previous findings^21^, confirms that the aberrant expression of PKCe in human platelets typifies the acute event. We demonstrated that FCM detection of platelet population expressing PKCe has a diagnostic feasibility, indeed PKCe-expressing PLTs shows a high sensitivity and specificity (87.5% and 84.4%, respectively) and a good diagnostic accuracy (AUC: 0.875), that is similar to cardiac troponin (AUC: 0.843), the marker of choice for serological diagnosis of AMI.

These data configure FCM detection of PKCe-expressing as potential diagnostic biomarker for AMI.

As compared to other several AMI biomarkers that have been recently described^25,26^, our study is characterized by two main novelties. First, the target of our assay, i.e. platelets, a cell population crucially involved in thrombus formation and, consequently, a pathophysiological marker complementary to the conventional marker of cardiac injury that has been historically the mainstay in clinical practice. Indeed, platelets’ genotypic and phenotypic abnormalities are not only a consequence of the acute event but also a leading cause of AMI^15,16^. More in details, we focused on platelets expressing PKCe, a disease-specific platelet protein, involved in the molecular pathways of platelet reactivity leading to platelet aggregation^27^. Furthermore, our previous finding demonstrated that PKCe is over-expressed both in mature and young platelets from AMI patients^21^, suggesting that this kinase could univocally characterize the platelet production wave of the peri-infarctual period, therefore detectable already at the onset of symptoms of myocardial infarction. The second novel aspect is the technique, i.e, an FCM-based assay that can be performed in 80 minutes, using a minimal amount of peripheral blood (5ul for each test), and without sample manipulation. Our proposed method configures as a fast, easy tool, amenable for standardization and clinical application in urgent setting.

Currently, high sensitivity troponin assay has improved speed and accuracy of clinical decision for CP patients at ED. Nevertheless, troponin is influenced by a multitude of clinical factors^26,28^. Consequently, the enhanced sensitivity is inevitably accompanied by a reduction in specificity^12^, which in turns leads to increasing cardiology consultations for a safe rule-out.

Given this scenario, we tested whether a biomarker involved in the pathophysiological mechanisms that cause AMI, i.e. PKCe-expressing PLTs, may add diagnostic benefit to the conventional biomarker of myonecrosis, i.e. cardiac troponin. The logistic regression model, combining cTnI and PKCe-expressing PLTs, reaches a very good diagnostic accuracy (AUC: 0.922), but it did not statistically improve diagnostic performance of cTnI. Interestingly, using simultaneously the two cut-off points as discriminating values, we found that only CP-AMI patients displayed both values that exceeded the cut-offs; conversely, only CP-noAMI patients showed both values under cut-off. This result has important clinical implications. Indeed, subjects with negative results for both troponin and PKCe-expressing PLTs represent the 68% (36/53) of our patient population, pointing out that AMI would have correctly been excluded at admission with only the first troponin and FCM assays in about two-third of patients. Furthermore, serial troponin assays, performed in 64.1% (34/53) of our population of could thus be limited to patients with positive results for either cTnI or PKCe-expressing PLTs, representing only the 24.5% of patients (13/53). Therefore, we highlighted a dual marker strategy potentially useful for stratification of patients presenting to ED with CP. Specifically: (i) the combination of positive results for both troponin and PKCe-expressing PLTs accurately identifies patients requiring urgent care for AMI; (ii) the combination of negative troponin and negative PKCe-expressing PLTs accurately identifies patients who can safely be discharged into outpatient care; (iii) positive results for either cTnI or PKCe-expressing PLTs identifies patients who need monitoring and serial blood sampling. Of note, this CP patient’s stratification can be performed with only one blood sampling at ED admission using fast assays.

Our study focuses on patients with acute coronary syndromes and myocardial necrosis, i.e. ST-segment elevation (STEMI) and non-ST segment elevation myocardial infarction (NSTEMI). Unstable angina (UA) was excluded in order to focus on patients having ischemic events considerable enough to lead to myocardial necrosis. However, if we focus on CP-noAMI patient group, a tendency towards increased amount of PKCe expressing platelets can be detected in UA group as compared to other CP-noAMI causes (Supplementary Fig. 3, panel d). The limited number of cases analyzed does not allow conclusions to be drawn.

Although we are aware that these data need to be validated in a larger cohort of patients, representative of STEMI and non-STEMI as well as of type-1 and type-2 MI, our results pave the way for a novel diagnostic strategy that has the potential to rapidly rule-out non-MI causes of chest pain. This is of utmost importance in the era of high sensitivity troponins, whose elevation may occur in several, no-MI conditions, such as arrhythmias, sepsis, myocarditis, trauma, poisonings, strenuous physical activity and many others. In this scenario, we propose a technique that may help corroborating alternative diagnosis and lead to a reduction of unnecessary hospital stay, improving cost-effectiveness of patient management.

## Methods

### Subjects

This study was performed according to the Declaration of Helsinki and the protocol was approved by the Ethical Committee of Parma University Hospital (Prot. no. 5344).

From March 2017 to July 2017, n. 94 consecutive patients admitted to the ED of the University Hospital of Parma with a complaint of chest pain were enrolled for this study. Exclusion criteria were represented by age <18 years and inability to release written informed consent.

The diagnostic workup was conducted by the emergency physicians according to the suggestions of the 2015 European Society of Cardiology (ESC) Guidelines for the management of acute coronary syndromes^9^. Diagnosis of AMI, STEMI and NSTEMI, was confirmed by a cardiologist according to the international clinical guidelines and universal definition^9,14^.

### Routine laboratory analysis

Laboratory parameters, including C-reactive protein, red blood cell distribution width (RDW), mean platelet volume (MPV), were measured immediately after blood withdrawal by standardized methods in the Laboratory of Clinical Chemistry and Hematology of Parma Hospital. Specifically, cardiac troponin I (cTnI) was measured at presentation (T1) and after 3 and 6 to 9 hours, as long as clinically indicated. cTnI was tested by Beckman Coulter Accu-TnI3 + assay perfomed on Unicell DxI 800 (Beckman Coluter, Brea. CA) analytical platform. This is a contemporary sensitive assay (i.e., capable to detect measurable values of cTnI in about 50% of healthy subjects) with detection limit of 0.01 ug/L, recommended by the manufacturer, and the diagnostic cut-off of 0.06 ug/L. The timing and treatment of patients were left to the discretion of the attending physicians. Patient data, laboratory test results and diagnosis at the time of ED discharge were collected from clinical records.

### Flow cytometry analysis of PKCe expressing platelets

After written informed consent, ~3.5 ml of peripheral blood were collected in 3.8% sodium citrate tubes for FCM analysis during routine laboratory tests. Samples processing and patients’ data collection were performed in blind and final diagnoses were unblinded only at the end of FCM data analysis.

For FCM analysis, 5 ul of whole blood was diluted 1:20 in PBS (Phosphate-Buffered Saline, Euroclone), and stained with 5 ul of anti-CD61-PE monoclonal antibody (Beckman Coulter) and 5 ul of anti-CD3-PeCy5 monoclonal antibody (Beckman Coulter) for 15′ at room temperature (RT). After incubation, sample was fixed adding 400 ul of Fixation Buffer (Biolegend) for 15′ at RT, and then 500 ul of Permeabilization Buffer (Biolegend) was added for an additional time of 15′ at RT. Finally, the sample was stained with 10 ul of anti-PKCe-FITC rabbit monoclonal antibody (code: NBP2–21823, Novus Biologicals) and, after 30′ of incubation at RT, it was analyzed by FC500 flow cytometer and the Expo ADC software (Beckman Coulter). As isotype control, the sample was stained with mouse IgG-PE, mouse IgG -PeCy5 and rabbit IgG-FITC.

Specifically, double staining with anti-CD61-PE and anti-PKCe-FITC monoclonal antibodies was performed on the entire patient’s population, while triple staining with anti-CD61-PE, anti-CD3-PeCy5 and anti-PKCe-FITC monoclonal antibodies was performed in a subset of 53 enrolled patients.

In double staining analysis, CD61^+^PKCe^+^ cells identified PKCepsilon-expressing platelets (PKCe-expressing PLTs) and the percentage of CD61^+^PKCe^+^cells was normalized according to CD61^+^ cells (Supplementary Fig. 6).

In triple staining analysis, lymphocytes were identified on whole blood cells according to CD3 expression. An immunological gate was constructed on CD3^-^ cell population, then PKCe-expressing PLTs has been detected as CD61^+^PKCe^+^ cells on CD3- population, and the percentage of CD3^−^ CD61^+^PKCe^+^cells was normalized for CD3^−^CD61^+^ cells (Supplementary Fig. 2).

### Statistical analysis

Continuous variables were represented as mean ± SD or median with interquantile range and compared using Student’s t test, Mann-Whitney test and chi-squared test as appropriate.

Correlation among continuous variables were assessed by Spearman-rank correlation coefficient. Logistic regression was used to combine PKCe-expressing PLTs with cTnI in the diagnosis of AMI.

Receiver-operating characteristic (ROC) curves were constructed to assess sensitivity and specificity throughout the value of PKCe-expressing PLTs (as CD61^+^PKCe^+^/CD61^+^ for double staining, or as CD3^−^ CD61^+^PKCe^+^/ CD3^−^CD61^+^ for triple staining), the concentration of cTnI, and their combination for diagnosis of AMI. Comparison of areas under the curve (AUC) was performed as recommended by Hanley *et al*.^29^.

Statistical analyses were performed using IBM SPSS statistics 25 and a p value < 0.05 was considered statistically significant.

### Ethical approval and informed consent statement

The authors stated that this study was performed according to the Declaration of Helsinki, all experimental protocols were approved by the Ethical Committee of Parma University Hospital (Protocol no. 5344). The authors confirmed that informed consent was obtained from all participants.

## Supplementary information

Supplementary information

## Data Availability

The authors stated that all data generated or analysed during this study are included in this published article and in its Supplementary Information files.
