# Supplementary material for: Combination of Platelet expression of PKCepsilon and cardiac troponin-I for early diagnosis of chest pain patients in the emergency department
Source: Sci Rep. 2019 Feb 14;9:2125. doi: 10.1038/s41598-019-38624-5 (PMC6375996; doi:10.1038/s41598-019-38624-5)
Supplement: Supplementary file 1 — Supplementary information [file 41598_2019_38624_MOESM1_ESM.pdf]

Supplementary Information for

Combination of Platelet expression of PKCepsilon and cardiac troponin-I for early diagnosis of chest pain patients in the emergency department.

Cecilia Carubbi[1], Elena Masselli[1], Giulia Pozzi[1], Maria Mattioli[2], Silvia Martini[3], Matteo Goldoni[1], Rosalia Aloe[4], Gianfranco Cervellin[5], Marco Vitale[1,6,7,*], Giuliana Gobbi[1,6].

[1]*Department of Medicine and Surgery, University of Parma, Parma, Italy.*

[2]*Division of Cardiology, Azienda Ospedaliero-Universitaria di Parma, Parma, Italy.*

[3]*Protein Phosphorylation Laboratory, Francis Crick Institute, London, United Kingdom.*

[4]*Dipartimento di Biochimica ad Elevata Automazione, Dipartimento Diagnostico, Azienda Ospedaliero-Universitaria di Parma, Parma, Italy.*

[5]*Emergency Department, Azienda Ospedaliero-Universitaria di Parma, Parma, Italy.*

[6]*Sport and exercise medicine center (SEM). University of Parma, Parma (Italy),*

[7]*CoreLab, Azienda Ospedaliero-Universitaria di Parma, University of Parma, Parma (Italy).*

***Correspondence to:** Marco Vitale, MD, marco.vitale@unipr.it, phone: +390521033034;

University of Parma, via Gramsci 14, 43126-PR, Italy

Supplementary figures and figure legends.

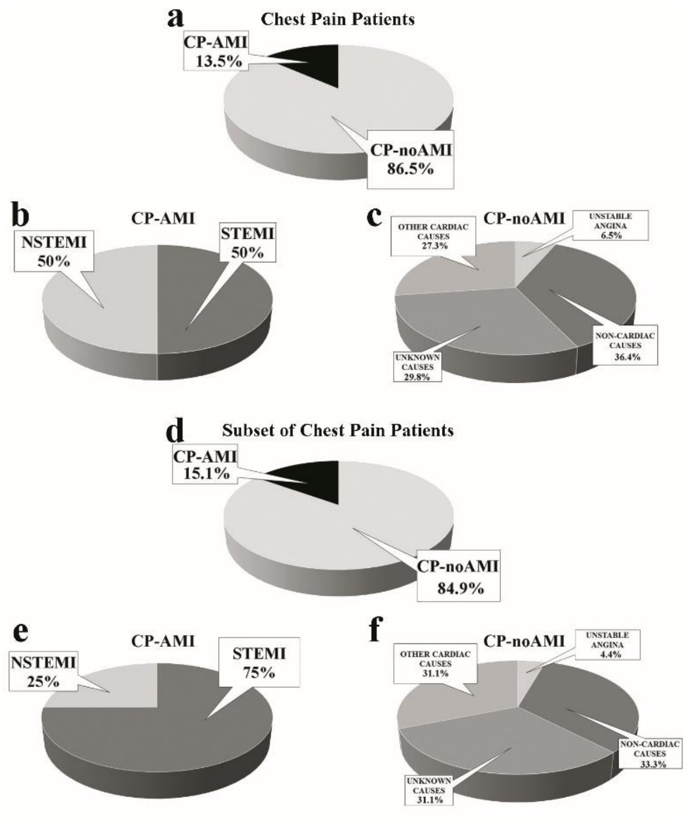

Figure S1. Differential diagnosis of enrolled chest pain patients. Panel a: percentage of chest patients with (CP-AMI) or without (CP-noAMI) final diagnosis of acute myocardial infarction in entire patient's population. **Panel b**: percentage of patients with final diagnosis of ST-segment elevation myocardial infarction (STEMI) and non-ST-segment elevation myocardial infarction (NSTEMI) in CP-AMI patients of the entire population. **Panel c**: percentage of patients with adjudicated final diagnoses of unstable angina, cardiac causes other than coronary artery disease, non-cardiac causes, and symptoms of unknown origin in CP-noAMI patients of the entire population. **Panel d**: percentage of chest patients with (CP-AMI) or without (CP-noAMI) final diagnosis of acute myocardial infarction in the subset of enrolled patients. **Panel e**: percentage of patients with final diagnosis of ST-segment elevation myocardial infarction (STEMI) and non-ST-segment elevation myocardial infarction (NSTEMI) in CP-AMI patients of the subset of enrolled patients. **Panel f**: percentage of patients with adjudicated final diagnoses of unstable angina, cardiac causes

other than coronary artery disease, non-cardiac causes, and symptoms of unknown origin in CP-noAMI patients of the subset of enrolled patients.

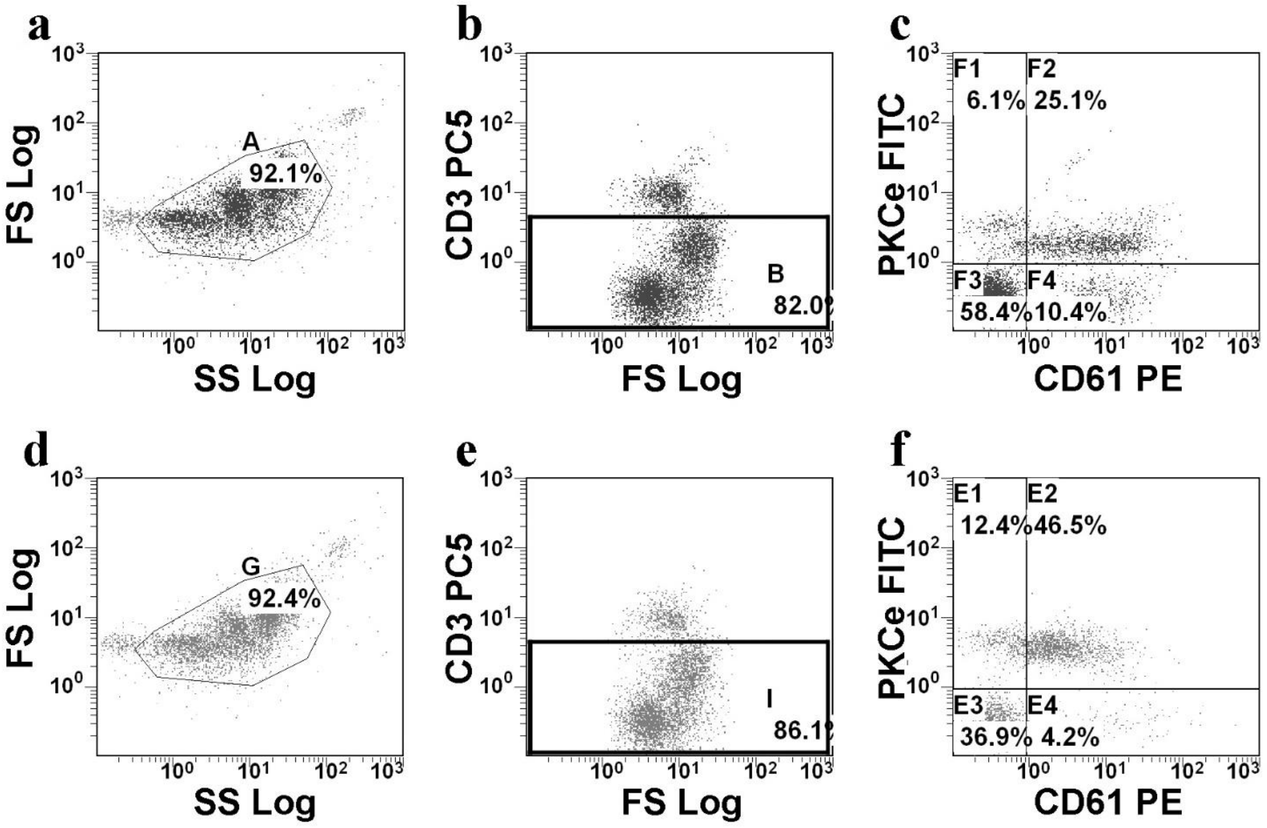

Figure S2. FCM detection of PKCe-expressing PLTs by triple staining. Flow cytometry gating strategy to analyse PKCe-expressing platelet, referred as $CD3^-CD61^+PKCe^+$ population, in a representative CP-noAMI patient (panels a-c) and CP-AMI patient (panels d-f). **Panel a and d:** morphological gate of whole blood cells referred to the forward scatter (FS Log) *vs* side scatter (SS Log) parameters. **Panel b and e:** CD3-PC5 expression on whole blood cells. Black rectangular gate identified $CD3^-$ population. **Panel c and f**: CD61-PE and PKCe-FITC expression on $CD3^-$ gated cells.

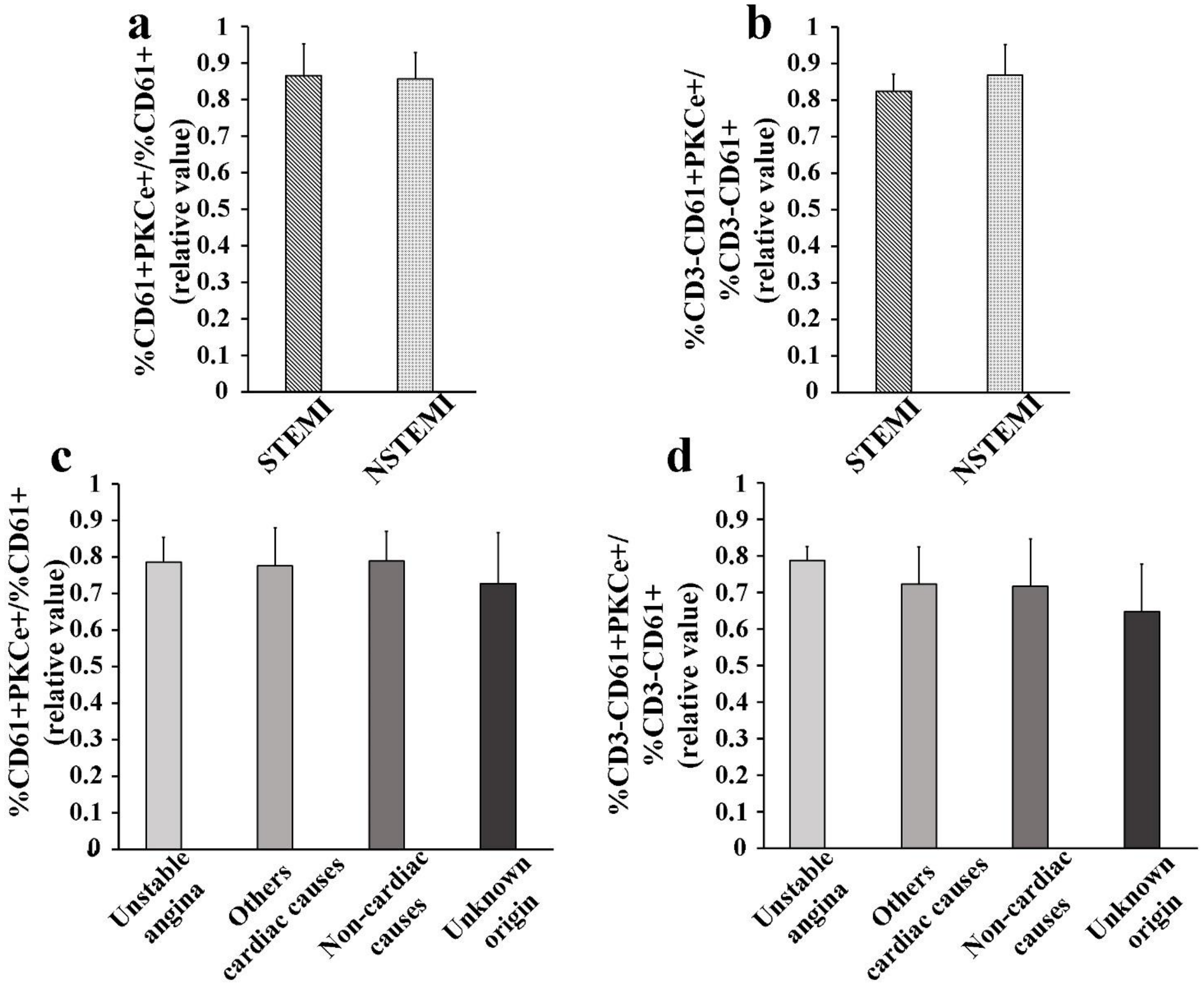

Figure S3. FCM analyses of PKCe-expressing PLTs. Panel a: PKCe-expressing PLTs in ST-segment elevation myocardial infarction patients (STEMI) (n.6 patients) and non-ST-segment elevation myocardial infarction patients (NSTEMI) (n.6 patients) of the entire patient population. PKCe-expressing PLTs are reported as $CD61^{+}PKCe^{+}$cell population and normalized according to $CD61^{+}$ cells. Data are reported as mean±SD. **Panel b:** PKCe-expressing PLTs in ST-segment elevation myocardial infarction patients (STEMI) (n.6 patients) and non-ST-segment elevation myocardial infarction patients (NSTEMI) (n.2 patients) in the subset of enrolled patients. PKCe-expressing PLTs are reported as $CD3^{-}CD61^{+}PKCe^{+}$cell population and normalized according to $CD3^{-}CD61^{+}$ cells. Data are reported as mean±SD. **Panel c:** PKCe-expressing PLTs in CP-noAMI patients of the entire population with adjudicated final diagnoses of unstable angina, cardiac causes other than coronary artery disease, non-cardiac causes, and symptoms of unknown origin. PKCe-expressing PLTs are reported as $CD61^{+}PKCe^{+}$cell population and normalized according to $CD61^{+}$ cells. Data are reported as mean±SD. **Panel d:** PKCe-expressing PLTs in CP-noAMI patients, of the subset of population, with adjudicated final diagnoses of unstable angina,

cardiac causes other than coronary artery disease, non-cardiac causes, and symptoms of unknown origin. PKCe-expressing PLTs are reported as $CD3^{-}CD61^{+}PKCe^{+}$cell population and normalized according to $CD3^{-}CD61^{+}$ cells. Data are reported as mean±SD.

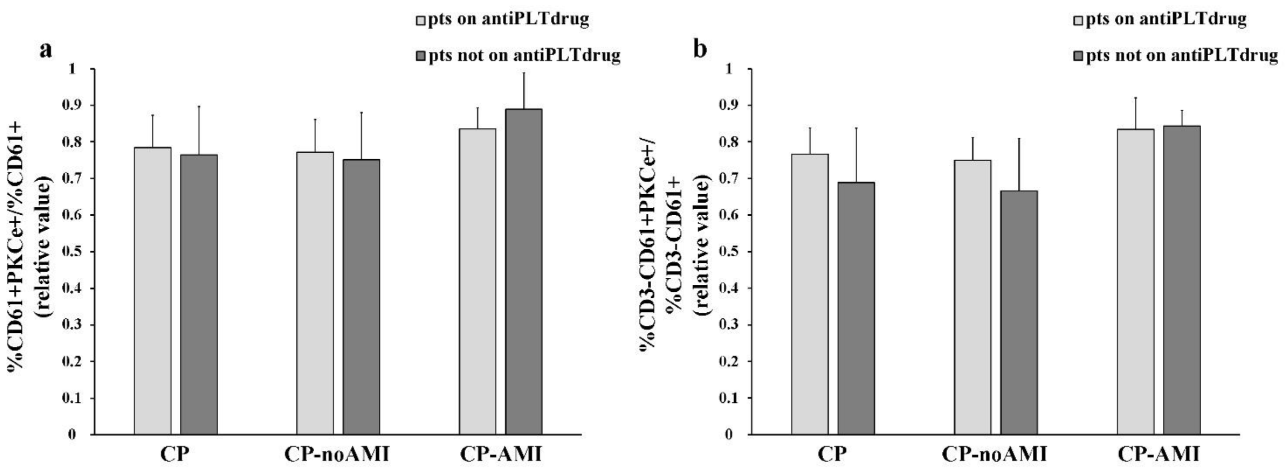

Figure S4. FCM analyses of PKCe-expressing PLTs. Panel a: PKCe-expressing PLTs in patients on anti-platelet drug (light gray) and in patients not on anti-platelet drug (dark gray) in the following patients groups: total Chest pain patients (CP), chest patients without (CP-noAMI) and with (CP-AMI) a final diagnosis of acute myocardial infarction. PKCe-expressing PLTs are reported as CD61+PKCe+ cell population normalized according to CD61+ cells. Data are reported as mean±SD. Not statistical differences were found in all comparison by Student t-test. **Panel b:** PKCe-expressing PLTs in patients on anti-platelet drug (light gray) and in patients not on anti-platelet drug (dark gray) in the following patients groups: total chest pain patients (CP), chest patients without (CP-noAMI) and with (CP-AMI) a final diagnosis of acute myocardial infarction. PKCe-expressing PLTs are reported as CD3-CD61+PKCe+cell population normalized according to CD3-CD61+ cells. Data are reported as mean±SD. Not statistical differences were found in all comparison by Student t-test.

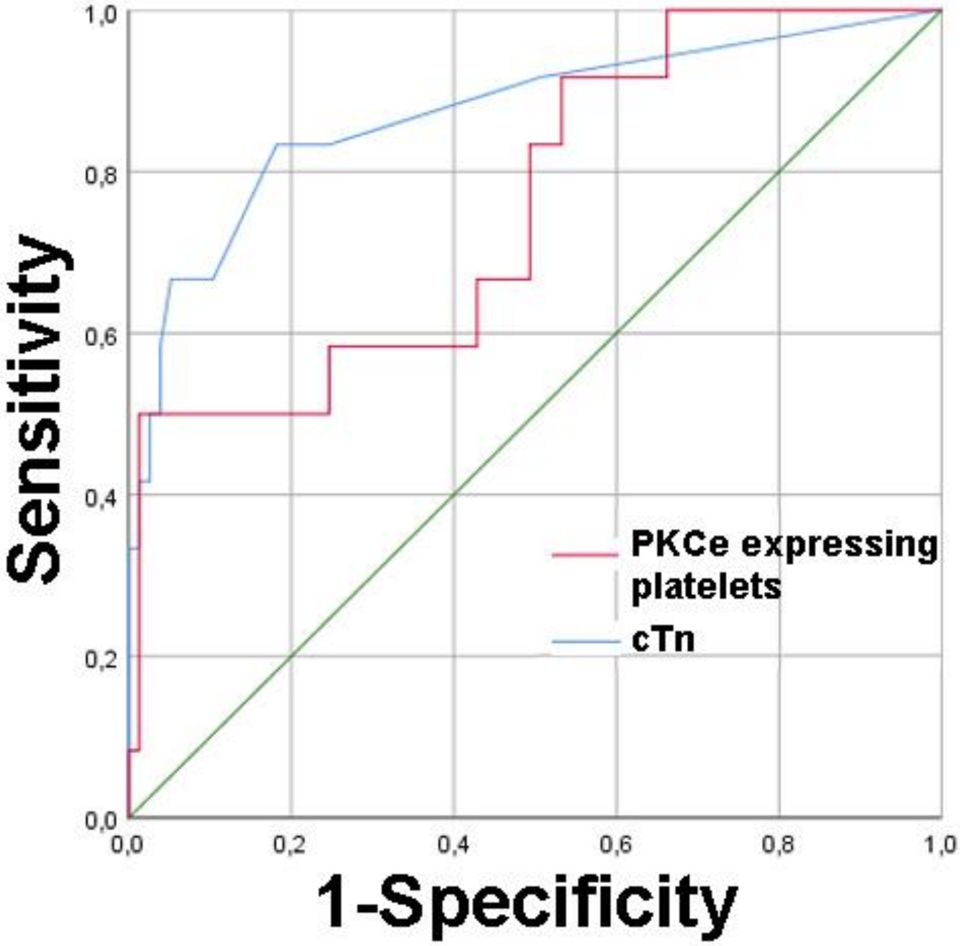

	AUC [95% CI]	Cut-off	Sensitivity	Specificity	PPV	NPV
CD61⁺PKCe⁺/CD61⁺	0.756 [0.606-0.907]	0.913	50%	98.7%	85.7%	92.6%
cTn	0.872 [0.743-1]	0.06	66.6%	93.5%	61.5%	94.7%

Figure S5. ROC curves of PKCe-expressing PLTs and cTn. ROC curves, AUC and CI, Cut-off, Sensitivity, specificity, PPV and NPV values for PKCe-expressing PLTs, analyzed as $CD61^+PKCe^+/CD61^+$ (red line), and cTn (blue line) in the entire patient population.

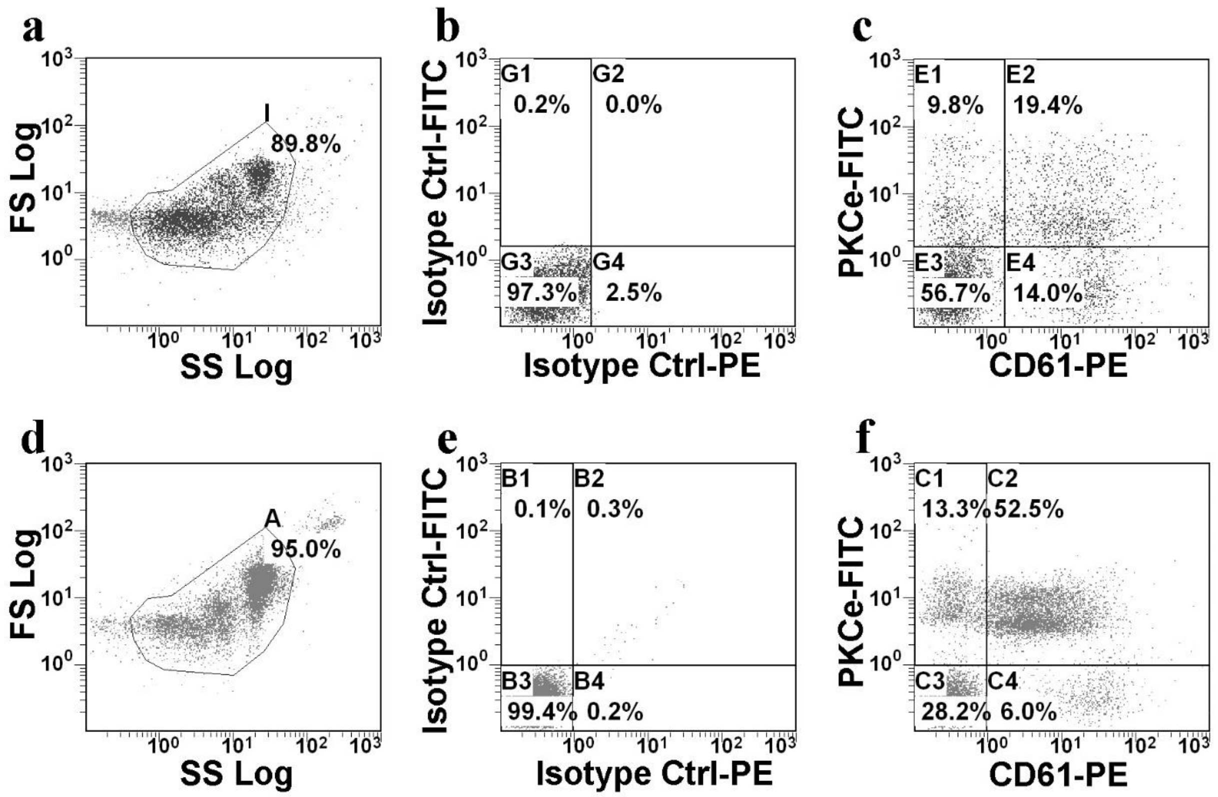

Figure S6. FCM detection of PKCe-expressing PLTs by double staining. Flow cytometry gating strategy to analyse PKCe-expressing platelet, referred as $CD61^{+}PKCe^{+}$ population, in a representative CP-noAMI (panels a-c) and CP-AMI (panels d-f). **Panel a and d:** morphological gate of whole blood cells referred to the forward scatter (FS Log) *vs* side scatter (SS Log) parameters. **Panel b and e:** Isotype control PE and FITC expression on gated whole blood cells. **Panel c and f:** CD61-PE and PKCe-FITC expression on gated whole blood cells.
